# α-Lipoic Acid Inhibits Apoptosis by Suppressing the Loss of Ku Proteins in *Helicobacter pylori*-Infected Human Gastric Epithelial Cells

**DOI:** 10.3390/nu14153206

**Published:** 2022-08-05

**Authors:** Dayong Park, Joo Weon Lim, Hyeyoung Kim

**Affiliations:** Brain Korea 21 FOUR Project, Department of Food and Nutrition, College of Human Ecology, Yonsei University, Seoul 03722, Korea

**Keywords:** α-lipoic acid, apoptosis, *Helicobacter pylori*, gastric epithelial cells, Ku protein

## Abstract

*Helicobacter pylori (H. pylori)* is a Gram-negative bacterium that colonizes the gastric mucosa and triggers various stomach diseases. *H. pylori* induces reactive oxygen species (ROS) production and DNA damage. The heterodimeric Ku70/Ku80 protein plays an essential role in the repair of DNA double-strand breaks (DSB). Oxidative stress stimulate apoptosis and DNA damage that can be repaired by Ku70/80. However, excessive reactive oxygen species (ROS) can cause Ku protein degradation, resulting in DNA fragmentation and apoptosis. α-lipoic acid (α-LA), which is found in organ meats such as liver and heart, spinach, broccoli, and potatoes, quenches free radicals, chelates metal ions, and reduces intracellular DNA damage induced by oxidative stress. Here, we investigated whether *H. pylori* decreases Ku70/80 and induces apoptosis, and whether α-LA inhibits changes induced by *H. pylori*. We analyzed ROS, DNA damage markers (γ-H2AX, DNA fragmentation), levels of Ku70/80, Ku–DNA binding activity, Ku80 ubiquitination, apoptosis indices (Bcl-2, Bax, apoptosis-inducing factor (AIF), and caspase-3), and viability in a human gastric epithelial adenocarcinoma cell line (AGS). *H. pylori* increased ROS, DNA damage markers, Ku80 ubiquitination, and consequently induced apoptosis. It also decreased nuclear Ku70/80 levels and Ku–DNA-binding activity; increased Bax expression, caspase-3 cleavage, and truncated AIF; but decreased Bcl-2 expression. These *H. pylori*-induced alterations were inhibited by α-LA. The antioxidant N-acetylcysteine and proteasome inhibitor MG-132 suppressed *H. pylori*-induced cell death and decreased nuclear Ku70/80 levels. The results show that oxidative stress induced Ku70/80 degradation via the ubiquitin–proteasome system, leading to its nuclear loss and apoptosis in *H. pylori*-infected cells. In conclusion, α-LA inhibited apoptosis induced by *H. pylori* by reducing ROS levels and suppressing the loss of Ku70/80 proteins in AGS cells.

## 1. Introduction

*Helicobacter pylori* (*H. pylori*) is a Gram-negative, microaerobic, spiral bacterium with a global prevalence [1]. In 2018, Zamani, et al. [2] reported that 44.9% of the world population was estimated to have been infected with *H. pylori* based on a meta-analysis of studies conducted from 2009 to 2016; however, the prevalence rate varies according to socioeconomic and geographic factors. *H. pylori* plays a pivotal role in the pathogenesis of various stomach diseases, ranging from gastritis to gastric cancer, by producing free radicals, including reactive oxygen species (ROS) and inflammatory cytokines [3,4,5]. Increased ROS levels in the gastric mucosa, caused by persistent *H. pylori* colonization [6], can lead to mutations and DNA damage, which can be predisposing factors for cancer [7,8].

ROS comprise endogenous factors that can damage DNA by oxidizing bases and causing double-strand breaks (DSBs) [9,10]. Persistent damaged DNA threatens genomic stability, which subsequently increases the likelihood of mutagenesis and carcinogenesis [11]. However, numerous pathways for repairing damaged DNAs, collectively known as DNA damage responses (DDRs), can repair base adductions, deletions, and single-strand breaks via base excision, nucleotide excision, and other pathways [12,13,14].

DSBs are repaired through homologous recombination (HR) and canonical non-homologous end joining (NHEJ) pathways [14,15]. The HR pathway uses adjacent homologous strands as templates to restore severed double strands, whereas after overhanging nucleotides are processed, the NHEJ pathway directly ligates damaged DNA ends [16]. Although both pathways can help to prevent chromosomal changes resulting from DSBs, the NHEJ pathway is more susceptible to local chromosome rearrangements than the HR pathway because of the absence of template strands [17,18].

The Ku70/80 heterodimer is essential for DSB repair via the NHEJ pathway [19,20]. After blocking damaged DNA ends, Ku forms a protein complex with the DNA-dependent protein kinase catalytic subunit (DNA-PKcs), Artemis, XRCC4, and ligase IV to mediate the consecutive NHEJ steps involved in DSB repair [21]. In addition, the Ku protein regulates cellular processes such as cell cycle arrest, telomere maintenance, and apoptosis [22]. The Ku70 protein can inhibit apoptosis by interacting with the pro-apoptotic protein Bax and blocking its translocation into the mitochondria [23,24,25]. However, when the intracellular DNA damage exceeds repair capacity, the expression of Ku70 and Ku80 is suppressed and apoptosis occurs [26,27,28,29,30]. We previously showed that oxidative stress decreases cell viability and Ku70 and Ku80 expression, indicating that such suppression plays an important role in apoptosis associated with excessive DNA damage [31]. When DSBs form, H2A histone family member X (H2AX) is phosphorylated to γ-H2AX, resulting in the formation of discrete γ-H2AX foci at DSBs [32].

α-lipoic acid (α-LA) is an organosulfur compound derived from octanoic acid. It contains two sulfur atoms connected by a disulfide bond. The carbon atom at c6 is chiral and the molecule exits as two enatiomers, (*R*)-(+)-LA and (*S*)-(-)-LA. Only (*R*)-(+)-LA (R-α-LA) exists in nature and is essential for aerobic metabolism [33]. Animal products such as red meat and organ meats are great sources of α-LA, but plant foods such as broccoli, tomatoes, spinach, and Brussels sprouts also contain it. [34]. In small amounts, α-LA can be enzymatically synthesized from octanoic acid in the mitochondria of humans [35]. α-LA plays an antioxidant role by quenching free radicals, chelating metal ions, and regenerating other antioxidants [36]. Owing to its powerful antioxidant properties, α-LA has recently been highlighted as a potential treatment for various diseases such as type-2 diabetes mellitus, Alzheimer disease, and many types of cancer [37,38]. α-LA protects human amniotic cells and DNA from hydrogen peroxide [39], and as well as the rat hippocampus against developing ethanol-induced DNA damage [40]. However, the precise mechanism through which α-LA protects cells against DNA damage remains unclear.

The present study aimed to determine whether *H. pylori* induces apoptosis by decreasing Ku70/80 proteins and whether α-LA inhibits *H. pylori*-induced DNA damage and apoptosis by reducing ROS levels and suppressing the loss of Ku70/80 in human gastric epithelial AGS cells.

We incubated AGS cells with antioxidant N-acetylcysteine (NAC) or the MG-132 proteasome inhibitor, and then stimulated them with *H. pylori* to determine the involvement of ROS and proteasomal degradation pathways in *H. pylori*-induced apoptosis.

For DNA markers and apoptosis indices, pro-apoptotic protein Bax, an anti-apoptotic protein Bcl-2, DNA fragmentation, caspase-3 cleavage, apoptosis inducing factor (AIF) activation, and H2AX focus formation were determined.

## 2. Materials and Methods

### 2.1. Cell line and Culture Conditions

Human gastric epithelial AGS cells (American Type Culture Collection, Rockville, MD, USA) were seeded in a complete medium comprising Roswell Park Memorial Institute (RPMI) 1640 medium (GIBCO, Grand Island, NY, USA) supplemented with 10% fetal bovine serum (FBS), 2 mM glutamine, 100 units/mL penicillin, and 100 µg/mL streptomycin (Sigma-Aldrich Corp., St. Louis, MO, USA), and were then cultured at 37 °C under a humidified 5% CO_2_ atmosphere.

### 2.2. Bacterial Strain and Growth Conditions

We cultivated the *H. pylori* strain NCTC 11637 (ATCC) on chocolate agar plates (Becton Dickinson Microbiology Systems, Franklin Lakes, NJ, USA) at 37 °C under microaerophilic conditions in an anaerobic chamber (BBL Campy Pouch^®^ System, Becton Dickinson Microbiology).

### 2.3. Reagents

α-LA (R-α-LA) (Sigma-Aldrich Corp.) was dissolved in ethanol (0.5 M) and stored under nitrogen gas at −20 °C (stock solution). NAC (A7280) and MG-132 (C2211, both from Sigma-Aldrich Corp.) were dissolved in distilled water and dimethyl sulfoxide (DMSO; Sigma-Aldrich Corp), respectively, and stored at −20 °C. NAC and MG-132 solutions were thawed and diluted in RPMI 1640 medium (GIBCO) to 1 mM and 0.5 µM. Control cells without MG-132 were incubated with DMSO. The amount of vehicle DMSO was less than 0.1%.

### 2.4. Infection of AGS Cells with H. pylori

AGS cells were cultured overnight to reach 80% confluence. *H. pylori* was harvested from chocolate agar plates and suspended in antibiotic-free RPMI 1640 medium supplemented with 10% FBS. For the infection ratio of *H. pylori* to AGS cells, multiplicity-of-infection (MOI) is used. MOI is the ratio of attached or infecting agents to susceptible targets.

To determine the optimal (MOI of *H. pylori* on cell viability, the cells were infected with *H. pylori* at a MOI of 20:1, 50:1, or 100:1 and cultured for 12 h and 24 h. To study the effects of α-LA on *H. pylori*-induced DNA damage and apoptosis, the cells were infected with *H. pylori* at a MOI of 100:1 and culture for 24 h.

### 2.5. Experimental Protocols

Prior to the studies on the effect of α-LA, the optimal duration of the stimulation on cell viability and apoptotic indices and the optimal MOI of *H. pylori* on cell viability and DNA fragmentation were determined.

For the studies on α-LA, the cells were treated with α-LA (10 or 20 µM) for 2 h, followed by stimulation with *H. pylori*. Control cells incubated without α-LA were incubated with ethanol (0.5 M). Intracellular ROS levels were determined after 2 h of *H. pylori* stimulation, whereas cell viability, apoptosis indices, DNA damage markers (γ-H2AX, DNA fragmentation), Ku70/80, Ku–DNA binding activity, and Ku80 ubiquitination were determined after 24 h.

To determine the involvement of oxidative stress and proteasomal Ku protein degradation in *H. pylori*-induced apoptosis, the cells were treated with antioxidant NAC and proteasome inhibitor MG-132 for 1 h, followed by stimulation with *H. pylori* for 24 h. The cell viability and levels of Ku70/80 were assessed in whole cell and nuclear extracts.

### 2.6. Preparation of Whole-Cell Extracts and Nuclear Extracts

Cells (1.0 × 10^6^/10 mL) were harvested using trypsin/EDTA, followed by centrifugation at 1000× *g* for 5 min. Cell pellets were resuspended in 100 μL buffer containing 10 mM Tris pH 7.4, 15 mM NaCl, 1% NP-40, and 1 tablet/50 mL protease inhibitor complex (Complete protease inhibitor; Roche, Mannheim, Germany), then lysed using a 1 mL syringe with several rapid strokes. The lysate was placed on ice for 30 min, followed by centrifugation at 13,000× *g* for 15 min. Supernatants were collected as whole-cell extracts. Nuclear extracts were prepared using an NE-PER Nuclear and Cytoplasmic Extraction Kit (Thermo Fisher Scientific Inc., Waltham, MA, USA), as described by the manufacturer. Briefly, the cells suspended in a cytoplasmic extraction reagent containing protease inhibitor complex were vortex-mixed for 15 s, then centrifuged at 13,000× *g* for 10 min. Nuclear pellets were resuspended in a nuclear extraction reagent, vortex mixed, and centrifuged at 13,000× *g* for 10 min. The supernatants were collected as nuclear extracts, the specificity of which was confirmed by identifying lamin B1 in the nuclear extracts. The protein concentrations were determined using Bradford assays (Bio-Rad Laboratories Inc., Hercules, CA, USA).

### 2.7. Measurement of Cell Viability and DNA Fragmentation

The cells (6.0 × 10^4^/1 mL) were cultured for 24 h. Viable cells were evaluated using 0.2% trypan blue exclusion (Sigma-Aldrich Corp.). DNA fragmentation was determined by assessing the nucleosome-bound DNA level in cell lysates as described previously [33]. DNA fragmentation, expressed as an enrichment factor, of unstimulated cells (None) was set at 1.

### 2.8. Measurement of Intracellular ROS Levels

The cells (2.0 × 10^5^/2 mL) were incubated with 10 µg/mL of dichlorofluorescein diacetate (DCFH-DA; Sigma-Aldrich Corp.) at 37 °C for 30 min, then DCF fluorescence (excitation and emission, 495 and 535 nm, respectively) was measured using a Victor5 multilabel counter (PerkinElmer Life and Analytical Sciences, Boston, MA, USA). The levels of ROS were quantified based on the relative increase in fluorescence. The intracellular ROS level of the untreated cells was set to 100%.

### 2.9. Electrophoretic Mobility Shift Assay (EMSA)

A Ku gel shift oligonucleotide (5′-GGGCCAAGAATCTTAGCAGTTTCGGG-3′) was labeled with [^32^P] dATP using T4 polynucleotide kinase (GIBCO). The end-labelled probe was purified from unincorporated [^32^P] dATP using a Bio-Rad purification column (Bio-Rad Laboratories Inc.) and recovered in a Tris-EDTA buffer. Nuclear extracts were incubated with a buffer containing ^32^P-labeled Ku oligonucleotides for 30 min and then resolved by electrophoresis on non-denaturing acrylamide gels. The gels were dried at 80 °C for 2 h and exposed to radiography film for 48 h at −80 °C.

### 2.10. Western Blotting

Whole-cell and nuclear extracts (20–50 µg protein/lane) were loaded onto 8–14% sodium dodecyl sulfate (SDS) polyacrylamide gels and separated by electrophoresis under reducing conditions. The proteins were electroblotted onto nitrocellulose membranes (Amersham Inc., Arlington Heights, IL, USA) and transfer was verified by reversible Ponceau S staining. Non-specific antigen binding on the membranes was blocked using 3% non-fat dry milk in Tris-buffered saline and 0.2% Tween 20 (TBS-T). The proteins of interest were detected using antibodies against Bcl-2 (sc-7382; dilution 1/500), Bax (sc-526; dilution 1/500), AIF (sc-13116; dilution 1/1000), Ku80 (sc-5280; dilution 1/3000), Ku70 (sc-5309; dilution 1/3000), and actin (sc-1615; dilution 1/3000) (all from Santa Cruz Biotechnology, Dallas, TX, USA), cleaved caspase 3 (9661s, Cell Signaling Technology, Danvers, MA, USA; dilution 1/500), caspase-3 (610322, BD Biosciences, San Jose, CA, USA; dilution 1/500), γ-H2AX (ab22551; dilution 1/1000), and lamin B1 (ab-16048, Abcam Cambridge, UK; dilution 1/1000) in TBS-T containing 3% dry milk and incubated overnight at 4 °C. The membranes were washed with TBS-T, then the primary antibodies were detected using horseradish peroxidase-conjugated secondary anti-mouse, anti-rabbit, and anti-goat antibodies. The proteins were visualized using Clarity Western enhanced chemiluminescence (ECL) Substrate (705061) and the ChemiDoc imaging system (Both from Bio-Rad Laboratories Inc.).

### 2.11. Immunofluorescence Staining

The cells (2.0 × 10^5^/2 mL) were fixed in 4% formaldehyde and incubated with a blocking buffer containing 1% BSA and 0.1% gelatin for 1 h, followed by anti-γ-H2AX antibody for 1 h. After three washes with PBS, the cells were incubated with rhodamine-conjugated goat anti-rabbit IgG antibody (Santa Cruz Biotechnology) for 1 h. The cells were washed again, incubated with 5 μg/mL 4′,6-diamidino-2-phenylindole (DAPI) in a blocking buffer for 30 min, and covered with Vectashield antifade medium. Images of the cells stained with rhodamine and DAPI were acquired using a laser scanning confocal microscope (Zeiss LSM 900; Carl Zeiss AG, Jena, Germany).

### 2.12. Co-Immunoprecipitation Assay

The cells (1.0 × 10^6^/10 mL) were lysed using an immunoprecipitation buffer containing 10 mM Tris-HCl (pH 7.4), 100 mM NaCl, 1 mM EDTA, 1 mM EGTA, 1 tablet/50 mL protease inhibitor complex (Complete protease inhibitor; Roche, Mannheim, Germany), 0.5% NP-40, 0.5% sodium deoxycholate, and 10% glycerol. The cells were then centrifuged at 15,000× *g* for 15 min. Anti-ubiquitin antibody (sc-8017) and protein A/G PLUS-agarose (sc-2003; both from Santa Cruz Biotechnology) were added to the clarified supernatant and incubated overnight at 4 °C. Protein G-antibody–antigen complexes were collected by four washes with an immunoprecipitation buffer. The pellet was resuspended and boiled for 10 min in 50 µL SDS sample buffer. Thereafter, the Ku80 expression was detected by Western blotting. Whole-cell extracts that were not incubated with the antibody and protein G/A were also Western blotted to detect the expression of Ku80 and actin as the input proteins.

### 2.13. Statistical Analysis

All of the data were statistically analyzed using one-way analysis of variance (ANOVA) followed by Tukey’s post hoc test and are reported as means ± standard error of three independent experiments (*n* = 4 per group). Values with *p* < 0.05 were considered statistically significant.

## 3. Results

### 3.1. H. pylori Induces DNA Fragmentation and Apoptosis in AGS Cells

**Figure 1:**
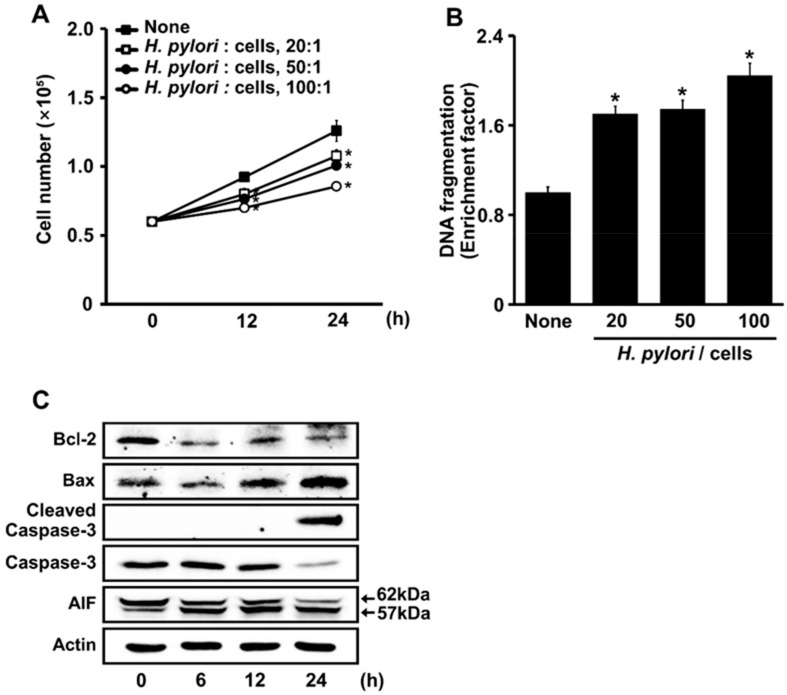
*H. pylori* increases the rates of cell death, DNA fragmentation, and apoptosis indices (Bcl-2, Bax, caspase-3, and AIF) in AGS cells. (**A**). The cells were stimulated without (■) or with ratios of *H. pylori* to bacteria of 20:1 (☐), 50:1 (●), and 100:1 (○), for up to 24 h. Viable cells were determined by trypan blue exclusion. All data are shown as means ± S.E. of three independent experiments. * p < 0.05 vs. unstimulated cells (None). (**B**) Cells were stimulated with *H. pylori* at indicated ratios of bacteria to cells for 24 h. DNA fragmentation, expressed as an enrichment factor, of unstimulated cells (None) was set at 1. All data are shown as means ± S.E. of three independent experiments. * p < 0.05 vs. unstimulated cells (None). (**C**) The cells were stimulated with 100:1 ratio of *H. pylori* to cells for indicated periods. Levels of Bcl-2, Bax, caspase 3, cleaved caspase 3, and truncated AIF in whole-cell extracts were determined by Western blotting. Actin was the loading control. AIF, apoptosis-inducing factor.

As shown in Figure 1A, the viable cell numbers of *H. pylori*-infected cells were lower than those of the uninfected cells (None) both at 12 h and 24 h of culture. At 12 h and 24 h, the viable cell numbers decreased depending on the MOI. For DNA fragmentation, cells stimulated with *H. pylori* at an MOI of 100 for 24 h showed a two-fold increase in the enrichment of DNA fragments compared with the unstimulated cells (“none”) (Figure 1B).

*H. pylori* increased the expression of Bax, and cleaved the activated caspase-3 and the caspase-independent apoptosis marker AIF. AIF was processed to a mature 62 kDa form and was *N*-terminally anchored to the inner mitochondrial membrane. During apoptosis, AIF was further processed and released into the cytosol as a truncated, 57 kDa soluble protein. Here, *H. pylori* infection increased truncated AIF, Bax, and cleaved caspase-3 (Figure 1C), but decreased the Bcl-2 in *H. pylori*-stimulated cells. These results suggest that *H. pylori* induced apoptosis via caspase-dependent and -independent pathways in AGS cells.

### 3.2. α-LA Inhibits H. pylori-Induced DNA Fragmentation and Apoptosis in AGS Cells

**Figure 2:**
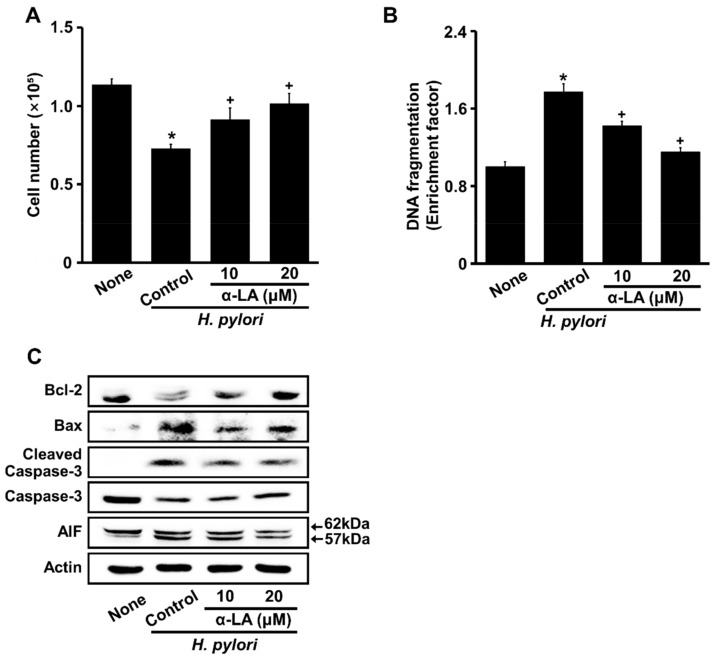
α-LA inhibits *H. pylori*-induced cell death, DNA fragmentation, and apoptosis indices (Bcl-2, Bax, caspase-3, and AIF) in AGS cells. The cells were incubated with indicated concentrations of α-LA for 2 h, and stimulated for 24 h with an *H. pylori* ratio to cells of 100:1. (**A**) Viable cells were evaluated by trypan blue exclusion. (**B**). DNA fragmentation, expressed as an enrichment factor, of unstimulated cells (None) was set at 1. All of the data are shown as means ± S.E. of three independent experiments. * p < 0.05 vs. unstimulated cells (None). ^+^ *p* < 0.05 vs. control cells (stimulated with *H. pylori* without α-LA). (C) Western blots of Bcl-2, Bax, caspase-3, cleaved caspase-3, and AIF proteins in whole-cell extracts. Actin was the loading control. AIF, apoptosis-inducing factor.

Figure 2A shows that α-LA dose-dependently inhibited *H. pylori*-induced cell death after 24 h of culture. *H. pylori* induced DNA fragmentation, which was suppressed by α-LA (Figure 2B). *H. pylori* increased Bax, cleaved caspase-3, and truncated AIF, but decreased Bcl-2 in AGS cells, all of which were inhibited by α-LA (Figure 2C). These results indicated that α-LA inhibited *H. pylori*-induced DNA fragmentation and apoptosis in AGS cells. 

### 3.3. α-LA Inhibits H. pylori-Induced Increases in ROS and γ-H2AX in AGS Cells

**Figure 3:**
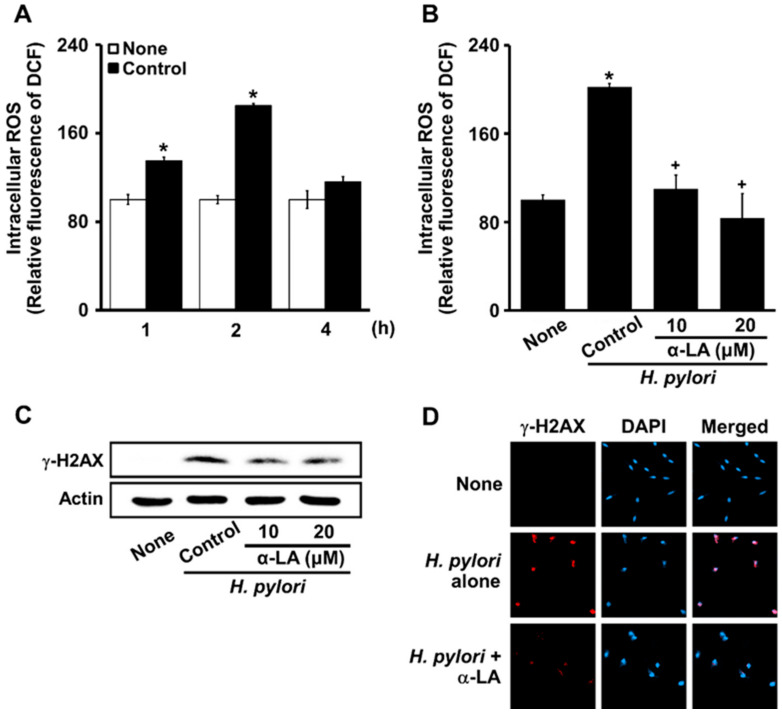
α-LA suppresses the *H. pylori*-induced increase in ROS levels, H2AX phosphorylation, and γ-H2AX foci formation in AGS cells. (**A**) Cells stimulated with an *H. pylori* ratio to cells of 100:1 for up to 4 h. The cells were incubated with α-LA at indicated concentrations for 2 h then stimulated with an *H. pylori* ratio to cells of 100:1 for 2 (**B**) and 24 (**C**,**D**) h. (**A**,**B**) ROS levels shown were determined using 2′,7′-dichlorofluorescin diacetate (DCFH-DA) assays. All of the data are presented as means ± S.E. of three independent experiments. * p < 0.05 vs. none (unstimulated cells without α-LA); ^+^ *p* < 0.05, vs. Control (cells stimulated by *H. pylori* without α-LA). (**C**) Western blots of γ-H2AX in whole-cell extracts. Actin was the loading control. (**D**) Cells and nuclei were stained with anti-γ-H2AX antibody (red) and DAPI (blue), respectively.

*H. pylori* increased ROS levels at 1 h, and even more at 2 h (Figure 3A). Thus, the effects of α-LA on ROS levels were determined in cells stimulated with *H. pylori* for 2 h (Figure 3B). α-LA decreased the levels of ROS and γ-H2AX increased by *H. pylori* (Figure 3C).

Using a confocal laser scanning microscope to visualize the intracellular γ-H2AX as the fluorescence intensity of rhodamine attached to an anti-γ-H2AX antibody, the status of γ-H2AX focus formation was determined. Cells stimulated with *H. pylori* formed robust γ-H2AX foci (Figure 3D, panel 2), which were decreased by α-LA (Figure 3D, panel 3). These results indicate that α-LA inhibited oxidative DNA damage via the antioxidant activity in the cells stimulated with *H. pylori*.

### 3.4. α-LA Inhibits H. pylori-Induced Decreases in Nuclear Ku70/80 in AGS Cells

**Figure 4:**
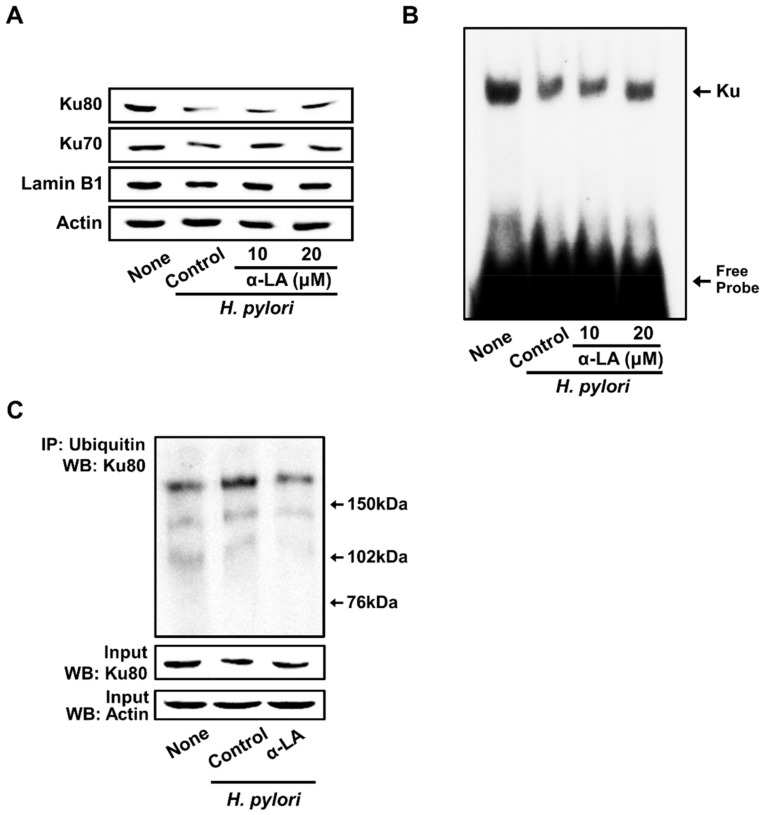
α-LA inhibits *H. pylori*-induced decreases in the nuclear Ku70/80, Ku–DNA binding activity, and ubiquitination of Ku80 in AGS cells. The cells were incubated with α-LA at the indicated concentrations for 2 h, then stimulated with an *H. pylori* to AGS cells ratio of 100:1 for 24 h. (**A)** Western blots of Ku70/80 in nuclear extracts. Actin and lamin B1 served as the loading control and nuclear marker, respectively. (**B**) The activity of Ku binding to DNA in the nuclear extracts was determined by an electrophoretic mobility shift assay. (**C**) Whole-cell extracts were immunoprecipitated (IP) with the anti-ubiquitin antibody, followed by Western blot analysis (WB) with anti-Ku80 antibody. The levels of the input proteins, Ku80 and actin, were determined by Western blot analysis. Input was used as the control for the protein expression.

Figure 4A shows that *H. pylori* decreased Ku proteins in the nuclear extracts. α-LA prevented a reduction in nuclear Ku70/80 induced by *H. pylori* in AGS cells (Figure 4A).

For determining the Ku–DNA binding activity, nuclear extracts were incubated with ^32^P-labeled Ku oligonucleotides, resolved by electrophoresis, and exposed to radiography film. As shown in Figure 4B, *H. pylori* decreased the Ku–DNA binding activity in the nuclear extracts, which was inhibited by the treatment of α-LA.

To determine the mechanism underlying the decrease in Ku, co-immunoprecipitation assays of the interactions between Ku80 and ubiquitin were performed. As shown in Figure 4C, *H. pyori* stimulation significantly increased the levels of ubiquitin-associated Ku80. Each ubiquitin molecule added ~8.5 kDa to the target protein Ku80, so an increased molecular weight of the protein was visible in the form of one or more higher molecular bands when stained with the Ku80 antibody. The results indicate that Ku80 is subject to poly-ubiquitination. The typical ‘‘ladder’’ of ubiquitinated Ku80 was higher than 150 kDa in size in the present study. *H. pylori*-induced ubiquitination of Ku80 was suppressed by α-LA.

### 3.5. Both NAC and MG-132 Inhibited Cell Death and Loss of Nuclear Ku Induced by H. pylori in AGS Cells

**Figure 5:**
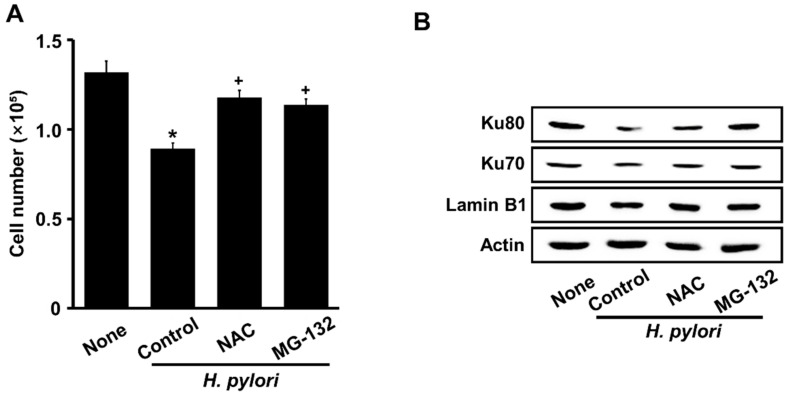
Both NAC and MG-132 suppress *H. pylori*-induced cell death and loss of nuclear Ku70/80 in AGS cells. Cells were incubated with 1 mM NAC or 0.5 μM MG-132 for 1 h, then stimulated with an *H. pylori* to AGS cells ratio of 100:1 for 24 h. (**A**) Viable cells were evaluated by trypan blue exclusion. All data are shown as means ±S.E. of three independent experiments. * p < 0.05 vs. None (unstimulated cells without NAC or MG-132). ^+^ *p* < 0.05 vs. Control (*H. pylori*-stimulated cells without NAC or MG-132). (**B**) Western blots of Ku70/80 in nuclear extracts with actin and lamin B1 as loading control and nuclear marker, respectively.

Figure 5A shows that NAC and MG-132 protected against cell death induced by *H. pylori* stimulation. Furthermore, a decrease in nuclear Ku70/80 induced by *H. pylori* was inhibited by NAC and MG-132 (Figure 5B). These results suggest that *H. pylori*-induced cell death and the loss of nuclear Ku70/80 are mediated by ROS and the ubiquitin-proteasome system in AGS cells.

## 4. Discussion

**Figure 6:**
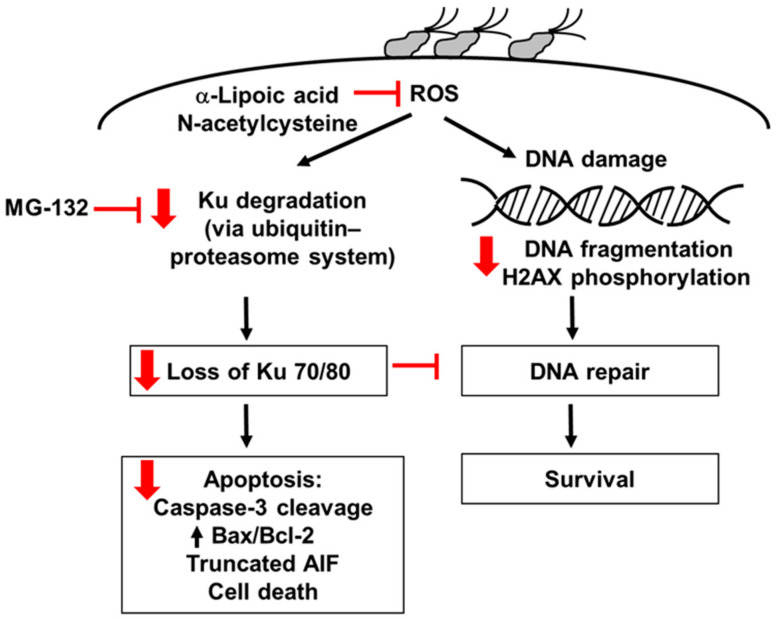
The proposed mechanism through which α-LA inhibits the loss of Ku proteins and apoptosis in *H. pylori*-infected cells. *H. pylori* increases ROS, which induces the degradation of Ku70/80 via the ubiquitin–proteasome system, resulting in the loss of nuclear Ku70/80 in *H. pylori*-infected cells. *H. pylori* increases oxidative DNA damage, determined by DNA fragmentation and H2AX phosphorylation in AGS cells. Loss of Ku proteins decreases the DNA repair process and induces apoptosis, assessed by increased Bax/Bcl-2, caspase-3 cleavage, truncated AIF, and cell death, in *H. pylori*-infected cells. α-LA reduces ROS levels and inhibits Ku degradation, loss of Ku70/80, DNA fragmentation, and apoptosis of *H. pylori*-infected cells. The antioxidant N-acetylcysteine and proteasome inhibitor MG-132 suppresses the degradation of Ku70/80 and apoptosis in *H. pylori*-infected cells. Red arrows represent the effect of α-LA. Red blocks mean “inhibition”. AIF, apoptosis-inducing factor; ROS, reactive oxygen species.

As summarized in Figure 6, the present study shows that *H. pylori* increases ROS, which induces the degradation of Ku70/80 via the ubiquitin–proteasome system, resulting in a loss of Ku70/80 in *H. pylori*-infected cells. *H. pylori* increases oxidative DNA damage, determined by the DNA fragmentation and H2AX phosphorylation in AGS cells. Loss of Ku proteins decreases the DNA repair process and apoptosis, assessed by increased Bax/Bcl-2, caspase-3 cleavage, truncated AIF, and cell death, in *H. pylori*-infected cells. α-LA reduces ROS levels and thus inhibits Ku degradation, loss of Ku70/80, DNA fragmentation, and the apoptosis of *H. pylori*-infected cells. The antioxidant NAC and proteasome inhibitor MG-132 suppresses the degradation of Ku70/80 and apoptosis in *H. pylori*-stimulated cells. The results show that oxidative stress might induce Ku70/80 degradation via the ubiquitin–proteasome system, causing a loss of Ku70/80 and apoptosis of the cells infected with *H. pylori*. α-LA inhibits the ROS-mediated degradation of Ku70/8 and DNA damage, which suppresses apoptosis in *H. pylori*-infected cells. Therefore, the dietary intake of α-LA-rich foods or the supplementation of α-LA may prevent *H. pylori*-associated gastric cancer development and progression.

*H. pylori* infection is correlated with the pathogenesis of gastric cancer [41]. We previously showed that ROS produced by NADPH oxidase increase the expression of interleukin-8 and mitochondrial dysfunction to propagate ROS production in infected gastric epithelial cells [42,43,44]. Biopsy specimens of gastric mucosa from patients infected with *H. pylori* revealed increased reactive oxygen metabolite levels [45]. As intracellular ROS accumulation induces DNA damage, oxidative stress can lead to apoptosis of gastric epithelial cells infected with *H. pylori* [46].

Extrinsic and intrinsic apoptotic stimuli upregulate the activity of pro-apoptotic Bcl-2 families such as Bax and Bak, and downregulate that of anti-apoptotic Bcl-2 families such as Bcl-2 and Bcl-XL. Proteins released from the intermembrane space of the mitochondria lead to the activation of caspase-3 [47,48]. Caspase-3 is the executioner of apoptosis, and its activated form has proteolytic activities against various important proteins, leading to chromatin condensation, cytoskeletal destruction, and DNA fragmentation [49,50]. However, caspase-3 is not responsible for apoptotic cell death, as evidenced by the finding that breast cancer cells with a deleted caspase-3 gene still undergo apoptosis in vitro [51,52]. Caspase-3 independent apoptotic pathways are mediated by released AIF from the intermembrane space of the mitochondria, which also results in DNA fragmentation [53].

We found that caspase-3 was cleaved after 24 h of *H. pylori* infection. However, cell death was already evident at 12 h. Therefore, *H. pylori*-induced apoptosis might involve a caspase-3-indendent apoptosis pathway, such as AIF activation. After 12 h of incubation, AIF was truncated after *H. pylori* infection, which might have been responsible for the decreased cell viability in the present study. These results agree with the findings that the levels of truncated AIF protein increase before caspase-3 activation in *H. pylori* stimulated gastric epithelial cells [54,55]. Taken together, these results indicate that an increase in ROS within 2 h of *H. pylori* infection results in the release of truncated AIF and the activation of caspase-3 in AGS cells.

For the expression analysis of the DDR proteins, *H. pylori* downregulates the gene expression associated with the repair of the base and nucleotide excisions and mismatches, and the HR pathway [56]. However, whether *H. pylori* downregulates the NHEJ pathway has remained unclear. It is evident that oxidative stress induces apoptosis and downregulates Ku70/80 and NHEJ proteins [28,29,30,31]. Ku70 is considered to play a vital role in the maintenance of chromosomal integrity and cell survival. The positive correlation between Ku70 and cancer [57] indicates that Ku70 is an important candidate target for anti-cancer drug development. Ku70 is able to suppress apoptosis by sequestering Bax in colon cancer cells. In addition, loss of Ku80 was found in colon cancer cells [58]. Epigallocatechin-3-gallate (EGCG)-induced apoptosis in lung cancer cells is mediated by decreased Ku70, and EGCG disrupts the interaction between Ku70 and Bax, which leads to an increased Bax expression in lung cancer cells [29]. These studies indicate the protective role of Ku70/80 against cancer cell apoptosis.

We previously found that ROS activates caspase-3, which induces Ku protein degradation and apoptosis in pancreatic acinar cells [30]. High levels of ROS activate caspase-3, resulting in a loss of Ku70/80 and apoptosis in gastric cancer cells [31]. These studies support the present finding showing that the loss of Ku proteins might be mediated by ROS and ubiquitin−proteasomes, leading to apoptotic cell death in *H. pylori*-infected cells.

As α-LA has a powerful antioxidant property, it reduces ROS and thus prevents ROS-mediated DNA damage in vitro and in vivo [39,40]. In addition, α-LA reduces the levels of 8-hydroxy-2′-deoxyguanosine (8-OH-dG), a biomarker of oxidative damage, in calf thymus models of oxidative DNA damage in vitro [59]. α-LA reduces oxidative stress and DNA damage in mice with ulcerative colitis [60]. Here, we found the mechanism through which α-LA protects cells against DNA damage. α-LA inhibits ROS-mediated activation of the ubiquitin–proteasome system and the degradation of Ku70/80 in *H. pylori*-infected cells. Therefore, α-LA prevents *H. pylori*-induced loss of Ku proteins and apoptosis in gastric epithelial cells.

In conclusion, α-LA inhibits *H. pylori*-induced apoptosis by reducing ROS levels and preventing Ku70/80 loss and DNA damage in gastric epithelial AGS cells. Supplementation with α-LA or the consumption of α-LA-rich foods may prevent *H. pylori*-induced gastric cancer development by preventing the loss of Ku proteins and apoptotic cell death.

## Data Availability

All data generated or analyzed during this study are included in this published article.

## Abbreviations

AGS	gastric epithelial adenocarcinoma cell line
α-LA	α lipoic acid
AIF	apoptosis-inducing factor
*H. pylori*	*Helicobacter pylori*
DAPI	4′,6-diamidino-2-phenylindole
DCF	dichlorofluorescein
DCFH-DA	dichlorofluorescein diacetate
DDRs	DNA damage responses
DMSO	dimethyl sulfoxide
DNA-PKcs	DNA-dependent protein kinase catalytic subunit
DSBs	double-strand breaks
ECL	enhanced chemiluminescence
EDTA	ethylene diaminetetraacetic acid
EGTA	ethylene glycol tetraacetic acid
EMSA	electrophoretic mobility shift assay
FBS	fetal bovine serum
HR	homologous recombination
H2AX	H2A histone family member X
MOI	multiplicity-of-infection
NAC	N-acetycysteine
NHEJ	non-homologous end joining
NP-40	nonidet P-40
8-OH-dG	8-hydroxy-2′-deoxyguanosine
PMSF	phenylmethylsulfonylfluoride
ROS	reactive oxygen species
RPMI	Roswell Park Memorial Institute
SDS	sodium dodecylsulfate
TBST	Tris-buffered saline and 0.2% Tween-20
